# Succinate metabolism: underlying biological mechanisms and emerging therapeutic targets in inflammatory bowel disease

**DOI:** 10.3389/fimmu.2025.1630310

**Published:** 2025-09-10

**Authors:** Mingzhu Dai, Shu Bu, Zhiwei Miao

**Affiliations:** ^1^ Department of Gastroenterology, Zhangjiagang TCM Hospital Affiliated to Nanjing University of Chinese Medicine, Zhangjiagang, China; ^2^ First Clinical Medical College, Affiliated Hospital of Nanjing University of Chinese Medicine, Nanjing, China

**Keywords:** succinic acid, inflammatory bowel disease, intestinal flora, SUCNR1, HIF-1α, immunometabolism

## Abstract

The global incidence of inflammatory bowel disease (IBD) continues to rise, yet its precise pathogenesis remains incompletely understood. In recent years, various gut microbiota-derived metabolites have been implicated in the development of IBD. Among them, succinic acid is a key metabolite produced by intestinal flora and serves as a central intermediate in the tricarboxylic acid (TCA) cycle, which plays a pivotal role in the IBD pathogenesis by modulating the intestinal mucosal barrier function, immune-metabolic reprogramming and cellular energy homeostasis. Abnormal succinate metabolism has also been linked to a range of metabolic disorders, including hepatitis, arthritis, diabetes mellitus, and cardiovascular diseases. Recently, its role in IBD has attracted growing interest. This review systematically elucidates the mechanisms by which succinate promotes pro-inflammatory immune phenotypes through a multifaceted network involving macrophage polarization, T-cell metabolic reprogramming, and epithelial-immune cell interactions, largely mediated via the SUCNR1 signaling axis. Furthermore, we explore the therapeutic potential of targeting succinate metabolism, offering new insights into IBD prevention and treatment.

## 1 Introduction

Inflammatory bowel disease (IBD) comprises ulcerative colitis (UC) and Crohn’s disease (CD). It is characterized by chronic, non-specific inflammation of the gastrointestinal tract. A recent epidemiological study reported a 47% increase in global IBD prevalence—from 3.32 million in 1990 to 4.9 million in 2019—with an alarming trend toward younger onset (1). The multifactorial etiology of IBD involves complex interactions among environmental triggers, genetic predisposition, and disruptions in the intestinal microbiome and immune balance. Modern lifestyle changes, including increased antibiotic use and dietary shifts (2, 3), have further elevated disease risk. The pathogenesis of IBD has not yet been fully elucidated, thus posing a challenge to the clinical cure of the disease. Current therapeutic options—such as aminosalicylic acid, corticosteroids, immunosuppressants, and biologics—can relieve symptoms, but there are limitations that long-term use may lead to infection and immune dysfunction. Therefore, a deeper understanding of IBD pathogenesis and identification of novel therapeutic targets is urgently needed. Among gut microbiota-derived metabolites, short-chain fatty acids, bile acids, and tryptophan derivatives have been extensively studied for their roles in IBD (4–6). It is worth noting that succinic acid, another important product of microbial fermentation, is playing a potential role in IBD. In a preliminary study on succinic acid levels in the stool of CD patients, Kaczmarczyk et al. found that active-phase CD patients exhibited elevated succinic acid concentrations (7). This suggests that succinate metabolism may play a key role in the pathogenesis of IBD.

SUCNR1 (GPR91), a succinate-specific G-protein-coupled receptor expressed on macrophages, dendritic cells, intestinal epithelial cells, kidneys, liver, and adipose tissue, engages succinate to launch pro-inflammatory cascades involving NF-κB and MAPK signaling (8), thereby serving as a metabolic checkpoint linking succinate accumulation to IBD pathogenesis (9, 10). Macias et al. demonstrated that Crohn’s disease patients exhibit elevated serum succinate with concomitant up-regulation of intestinal SUCNR1; *in vitro* studies further confirmed that this axis drives mucosal inflammation and fibroblast activation (11). As both a normal intermediate in the TCA cycle and an overproduced metabolite by gut bacteria like Enterococcus faecalis, succinate demonstrates antioxidant and metabolic-promoting functions at physiological concentrations. However, abnormal accumulation of succinate during hypoxia, microbial imbalance, or mitochondrial dysfunction can trigger immune dysregulation and cellular energy metabolism disorders (12, 13). For instance, intestinal bacteria-generated succinate activates macrophages via the SUCNR1-HIF-1α pathway, enhances glycolysis, and leads to cytokine imbalances such as IL-1β elevation, thereby exacerbating neonatal necrotizing enterocolitis and ulcerative colitis (14). Conversely, limiting microbial succinate production or reshaping the gut microbiota can promote regulatory T cell differentiation and alleviate symptoms (15).

In summary, succinate’s influence on IBD encompasses immune activation, energy metabolism disruption, and inflammatory signaling pathway modulation. This review aims to comprehensively outline the biological mechanisms by which succinate contributes to IBD pathogenesis and discusses emerging therapeutic strategies targeting succinate metabolism, providing a novel perspective for future research and clinical intervention.

## 2 Sources of succinic acid

### 2.1 Exogenous succinic acid: a metabolic source for gut flora

In sterile tissues, succinic acid is mainly derived from the tricarboxylic acid cycle, but the distal gastrointestinal tract with dense microorganisms can also produce succinic acid as a byproduct through anaerobic nutritional fermentation (16, 17). Studies have shown that succinate is nearly undetectable in the feces of germ-free (GF) mice compared to conventionally raised mice, indicating that gut microbiota are the major contributors to intestinal succinate production.Typically, the phylum Bacteroidetes, the phylum Firmicutes, and the genus Actinobacteria are the major intestinal producers of succinate, including Mycobacterium spp, Mycobacterium fragilis, Prevotella spp, Mycobacterium avium, Mycobacterium parvum, Lactobacillus plantarum, Bifidobacterium animalis, and Bifidobacterium paratuberculosis (18–25). In this, succinic acid concentrations typically range between 1 and 3 mmol/L (or mmol/kg), but the exact values may vary depending on species and sample type (26–28). Experiments analyzing cecal metabolites in GF mice colonized with dominant bacterial strains from neonatal and adult mice showed significantly elevated succinate levels in GF mice colonized with Bacteroides pimplei compared to uncolonized controls (29–31). Similarly, De Vadder et al. demonstrated that in mice colonized by succinic acid producers, increased levels of cecal succinate were found in conventional mice colonized by Prevotella (32); Supporting these animal studies, Sanderson et al. detected abundant DNA from succinate-producing Mycobacterium avium subspecies paratuberculosis on the inflamed intestinal mucosa of Crohn’s disease (CD) patients (28).

The gut microbiota tightly controls luminal succinate levels through a dynamic “production-consumption” balance. Under healthy conditions, Firmicutes such as Clostridium spp., Succinivibrio dextrinosolvens (18, 33, 34), and Burkholderia actively consume succinate and convert it into propionate, keeping the intraluminal concentration low (17, 33, 35). In inflammatory bowel disease (IBD) and Crohn’s disease patients, as well as in multiple chemically induced colitis models (22, 36), this balance is disrupted: on the one hand, succinate-producing taxa—particularly within the Bacteroidetes phylum—expand markedly (37, 38); on the other hand, the above-mentioned succinate-utilizing bacteria decline. Metagenomic analyses further demonstrate that the loss of specific succinate-consuming strains is the key driver of pathologic succinate accumulation (39). Microbiota-driven succinate build-up not only acts as a pro-inflammatory signal but can also directly exacerbate intestinal inflammation, as shown in “patient-microbiota-to-germ-free-mouse” colonization experiments (40–42).

Importantly, microbiota-derived succinate is not merely a pro-inflammatory metabolite; it also plays a central role in systemic energy homeostasis. Succinate absorbed by intestinal epithelial cells serves as a substrate for intestinal gluconeogenesis (IGN). The newly synthesized glucose is released into the portal vein and reaches the liver (43), lowering fasting glycemia while, via the vagus-nerve–brain axis, enhancing satiety and reducing food intake (43, 44). Thus, dysbiosis not only triggers local inflammation through succinate accumulation, but may also disturb the IGN-mediated metabolic–neural feedback loop, further compromising host energy balance.

### 2.2 Endogenous succinic acid: a source of host cell synthesis

Succinate is a pivotal intermediate of the host mitochondrial tricarboxylic acid (TCA) cycle. Under aerobic conditions, acetyl-CoA and oxaloacetate are converted to succinate through the canonical oxidative branch, catalyzed sequentially by citrate synthase, aconitase, isocitrate dehydrogenase, and succinyl-CoA synthetase. Subsequently, succinate dehydrogenase (SDH) oxidizes succinate to fumarate, concomitantly generating FADH_2_. Because SDH also functions as complex II of the mitochondrial electron transport chain, this reaction not only completes a step of the TCA cycle but also directly couples the cycle to oxidative phosphorylation (45). When oxygen tension drops, the TCA cycle can run in reverse: phosphoenolpyruvate carboxylase (PPC) (46), phosphoenolpyruvate carboxykinase (PCK), pyruvate carboxylase (PYC) (47), or malic enzyme (MAE) (48) drive the reductive branch, causing the oxaloacetate–malate pool to accumulate succinate in the reductive direction (49, 50). Beyond these “central routes,” an additional ~18% of succinate in inflammatory microenvironments is generated via the γ-aminobutyric acid (GABA) shunt from glutamine (6): glutamine is first converted to GABA, which is then metabolized in two steps by GABA transaminase and succinic-semialdehyde dehydrogenase to yield succinate (51). Moreover, after the malate–aspartate shuttle or purine-nucleotide shuttle elevates mitochondrial fumarate levels (52, 53), the reverse activity of SDH can reduce fumarate back to succinate (54), establishing a reversible “overflow” pathway.

Succinate synthesized in mitochondria exits via the dicarboxylate carrier into the cytosol and is then released into the extracellular space through voltage-dependent anion-selective channels (VDAC). In the cytosol, it can also be secreted into the extracellular milieu in a pH-dependent manner via organic anion/dicarboxylate transporters or monocarboxylate transporters. Extracellular succinate acts in an autocrine or paracrine fashion to activate the widely expressed SUCNR1 receptor in tissues such as the intestine and liver, thereby enhancing intestinal gluconeogenesis, increasing insulin sensitivity, and optimizing lipid metabolism. Thus, succinate functions not only as a nodal molecule linking the TCA cycle to oxidative phosphorylation but also as a signaling metabolite that conveys metabolic states across cells and organs.

### 2.3 Synergistic action of endogenous and exogenous succinate: dose-dependent regulation of IBD by the succinate receptor

Exogenous succinate, primarily derived from metabolic activities of gut microbiota, regulates intestinal barrier function, immune responses, and inflammatory processes by binding to host receptors SUCNR1. It may also enter the bloodstream to exert systemic effects, serving as a key mechanism in host-microbiome interactions. In contrast, endogenous succinate, acting as a tricarboxylic acid cycle intermediate, functions as the core of energy metabolism and is typically confined within cells. However, under conditions of hypoxia, inflammation, or mitochondrial stress, it accumulates intracellularly, stabilizing HIF-1α and activating inflammatory signaling pathways. Despite differences in origin and mechanisms, both exogenous and endogenous succinate can synergistically promote disease progression in pathological states like IBD at both local and systemic levels. Specifically, microbial dysbiosis elevates intestinal succinate levels (5-8 mM in IBD patients vs. 1-3 mM in healthy individuals), exacerbating local inflammation. Notably, changes in succinate concentration produce a biphasic effect through SUCNR1 receptors: physiological concentrations <10 μM promote epithelial repair, while concentrations>100 μM induce IL-1β release and M1-type macrophage polarization via the SUCNR1/Gq pathway, thereby driving inflammation (55). This dose-dependent effect has been mechanistically validated: *In vitro* transfection models demonstrate that succinate concentrations in the nanomolar to millimolar range can dose-dependently activate SUCNR1-mediated Gi and Gq signaling, thereby regulating NFAT/SRE transcription reporter gene activity. Crucially, within physiologically relevant concentrations, this effect sufficiently alters the SUCNR1-dependent immune gene expression profile in M2 macrophages, with its effects being counteracted by SUCNR1 receptor antagonists or specific Gq inhibitors. Consequently, physiological fluctuations in succinate concentration can dynamically modulate downstream functions through SUCNR1’s dose-response curve (56). However, the interplay between endogenous and exogenous succinate in synergistic regulation remains to be elucidated. In conclusion, investigating the distinct biosynthetic sites and mechanisms of exogenous versus endogenous succinate is essential for understanding the pathogenesis of related metabolic disorders and developing targeted precision interventions targeting the succinate pathway.

## 3 Succinic acid affects intestinal immune mechanisms

### 3.1 Succinate-mediated regulation of the intestinal epithelial barrier

In pathological conditions such as inflammation and cancer, succinate abnormally accumulates, indicating its potential role as a critical mediator in the interaction between gut microbiota and the host’s intestinal immune system. Mechanistically, succinate primarily regulates intestinal barrier function and immune responses by modulating intestinal epithelial cells and immune cells. Intestinal epithelial cells (IECs) are central regulators of gut immunity and encompass key subtypes such as Paneth cells, tuft cells, and goblet cells. Paneth cells serve as sentinels of the intestinal crypts by secreting antimicrobial peptides and sustaining epithelial barrier function (57). Their differentiation depends on the transcription factor Sox9. During mouse embryogenesis, genetic inactivation of Sox9 in intestinal epithelial cells results in the loss of Paneth cells and impaired antimicrobial-peptide production, leading to subtle shifts in the gut microbiota—most notably the overgrowth of succinate-producing taxa like Prevotella and Bacteroides. This dysbiosis elevates luminal succinate levels and exacerbates intestinal inflammation (58). In the inflamed intestines of Crohn’s disease patients, Paneth cells are similarly altered, the microbiota is dysbiotic, and succinate accumulates (40, 59).

Tuft cells are key epithelial mediators that orchestrate type 2 immune responses of the mucosa against eukaryotic parasites—including helminths and protozoa—and participate in central facets of chronic inflammatory dysregulation. Succinate activates tuft-cell taste-chemosensory signaling via the receptor SUCNR1 (60–62). SUCNR1-dependent epithelial tuft cells initiate a positive-feedback loop by secreting IL-25, which activates type 2 innate lymphoid cells (ILC2s) to produce the type 2 cytokine IL-13. This, in turn, drives intestinal type 2 immunity and increases the number of Paneth cells that promote dysbiosis and chronic inflammation (60, 63–65). Together, these findings suggest that crosstalk between Paneth and tuft cells may cooperatively modulate gut microbial homeostasis and the inflammatory status of the intestinal mucosa. Mechanistically, the SUCNR1 receptor on tuft cells acts as a molecular bridge linking luminal succinate sensing to downstream immune and epithelial effector programs, particularly under conditions of compromised Paneth-cell function.

Interestingly, other studies have shown that exogenous or intracellularly accumulated succinate can foster mucosal repair via short-term homeostatic modulation (66). Succinate derived from the microbiota or the diet enhances goblet-cell differentiation and strengthens the mucosal barrier through SUCNR1 activation, thereby attenuating inflammation and restoring microbial homeostasis (67, 68). Moreover, dietary succinate alone is sufficient to elicit type 2-immune-mediated goblet-cell restoration and to improve both mucosal-barrier integrity and microbial dysbiosis; in SUCNR1-deficient mice, however, a succinate-enriched diet fails to augment intestinal barrier function.

### 3.2 Succinate-mediated modulation of T cell differentiation

Succinate plays a pivotal role in modulating T-cell function within the inflammatory microenvironment by reshaping energy metabolism and signaling pathways. During T-cell activation, both the gene expression and enzymatic activity of succinate dehydrogenase (SDH) rise sharply. Conversely, SDH deficiency curtails T-cell survival and proliferation. In T cell-driven mouse and human colitis models, loss of the SDH subunit SDHA in intestinal epithelial cells (IECs) reprograms T-cell metabolism—manifested as succinate accumulation, heightened glycolysis, mitochondrial dysfunction, and excessive ROS production. This metabolic shift not only drives T cells from oxidative phosphorylation toward a glycolytic, pro-inflammatory program but also compromises their anti-inflammatory capacity, thereby disrupting IEC oxidative phosphorylation and further elevating local succinate levels (65, 69). Accumulated succinate has been shown to stabilize HIF-1α, thereby promoting CD4^+^ T-cell differentiation toward the Th17 lineage while suppressing regulatory T-cell (Treg) generation, thus amplifying inflammation (40, 70). Importantly, an elevated succinate/α-ketoglutarate (AKG) ratio serves as a key trigger for Th17 polarization, a process that can be partially reversed by restoring AKG levels or reducing succinate accumulation (71). Succinate also influences T-cell function via its specific receptor SUCNR1; receptor activation augments pro-inflammatory T-cell proliferation and markedly increases secretion of pro-inflammatory cytokines such as IL-6 and IL-17, whereas these effects are markedly attenuated in SUCNR1-knockout mice (72). Likewise, T cells lacking the SDH subunit SDHB exhibit an increased succinate/AKG ratio, induce pro-inflammatory gene signatures, and foster differentiation of pro-inflammatory T-cell subsets (73).

Studies have revealed that the TCA cycle exerts a dual role in metabolically adaptive CD8^+^ T cells: on one hand, it supplies conventional metabolic intermediates; on the other, it actively secretes succinate as an autocrine signal. Succinate, by engaging the pro-inflammatory receptor SUCNR1, directly drives inflammatory cytokine synthesis and reinforces cytotoxicity (72). More importantly, during mitochondrial oxidation of succinate, reverse electron transport (RET) generates large amounts of reactive oxygen species (ROS). These ROS act as second messengers to further amplify pro-inflammatory gene expression, thereby completing metabolic reprogramming (74) and establishing a key hub for initiating T-cell effector functions.

Although research on succinate and T cells is still rapidly expanding, the existing evidence strongly supports its influence on adaptive immunity. Succinate can indirectly shape T-cell responses by modulating dendritic-cell (DC) function. Via activation of SUCNR1, succinate augments DC migration and antigen-presenting capacity, thereby promoting antigen-specific T-cell activation. These effects are particularly pronounced under inflammatory conditions and contribute to both local and systemic immune responses. Knocking down SUCNR1 markedly attenuates these outcomes, underscoring its role in inflammatory regulation (75, 76). In rheumatoid-arthritis (RA) patients, extracellular succinate that accumulates in synovial fluid engages SUCNR1 to direct DC migration to lymph nodes, leading to an increased frequency of Th17 cells. Consequently, neutrophil infiltration into joints that exacerbates inflammation (77).

Succinate sits at the nexus of the network of gut microbiota, host metabolism, and immune interactions. Through its two central molecular hubs—the SUCNR1 receptor and the SDH enzyme—it exerts intricate, often bidirectional control over intestinal epithelial barrier integrity, innate immunity (macrophage polarization, tuft-cell activation), and adaptive immunity (T-cell differentiation). Collectively, these studies not only illuminate the multifaceted roles of succinate in governing T-cell differentiation and function, but also—by revealing its connections to the Th17/Treg balance, HIF-1α signaling, and metabolic pathways—offer potential therapeutic targets and directions for the treatment of inflammatory diseases.

### 3.3 Succinate-mediated coordination of macrophage M1/M2 polarization and energy metabolism

Macrophage polarization is a critical determinant of intestinal inflammation, particularly in ulcerative colitis (UC), where the balance between pro-inflammatory M1 and anti-inflammatory M2 phenotypes governs disease progression and resolution (78). Succinate has emerged as a pivotal regulator of this polarization process, influencing both intracellular and extracellular signaling mechanisms. During intestinal inflammation, macrophages—upon stimulation with the Toll-like receptor 4 (TLR4) ligand lipopolysaccharide (LPS)—shift from oxidative metabolism to aerobic glycolysis and the pentose phosphate pathway while simultaneously downregulating fatty-acid oxidation and OXPHOS (79, 80), undergoing metabolic reprogramming. This ultimately leads to the accumulation of succinate (81–83).

Accumulated succinate exacerbates M1-M2 polarization imbalance, amplifying inflammatory cascades. Two key mechanisms determine its inflammatory phenotype: mitochondrial reactive oxygen species (ROS) generation from succinate oxidation is crucial. While succinate is oxidized to fumarate via succinate dehydrogenase (SDH), the electrons derived from this oxidation reduce the Coenzyme Q (CoQ) pool. Under conditions of a high mitochondrial membrane potential, electrons can flow backwards (reverse) from the reduced CoQ pool into complex I. This process of reverse electron transport (RET) drives ROS production at complex I, promoting macrophage polarization to the M1 subtype (84–86).

However, the succinate–SUCNR1 axis plays a pivotal role in M2 macrophage polarization and the resolution of inflammation (87, 88). Intriguingly, although succinate is produced by pro-inflammatory macrophages, its receptor SUCNR1 is predominantly expressed on anti-inflammatory (M2) macrophages (55, 89). Recent studies have underscored the anti-inflammatory function of SUCNR1. Park et al. demonstrated that succinate-treated macrophages can attenuate colitis in mice (90). Specifically, intracellular accumulation of succinate in adipose-derived mesenchymal stem cells (MSCs) triggers the release of PGE_2_—a soluble mediator that suppresses macrophage inflammatory responses and drives their transition from the M1 to the M2 phenotype (91). Moreover, engagement of SUCNR1 on the surface of anti-inflammatory macrophages by extracellular succinate reinforces this phenotypic switch via Gq- and PLC-dependent signaling pathways (56). Notably, studies have shown that a high α-ketoglutarate (α-KG)-to-succinate ratio favors the activation of anti-inflammatory macrophages, whereas a low ratio promotes pro-inflammatory activation (71, 92). This underscores how metabolic balance critically determines immune-cell fate.

Moreover, succinate orchestrates metabolic reprogramming in macrophages via the HIF-PKM2 axis, overriding homeostatic glucose metabolism to drive a glycolytic shift. Succinate, acting as an endogenous danger signal, accumulates inside the cell and blocks HIF-1α hydroxylation-dependent degradation by inhibiting prolyl hydroxylase activity. This stabilizes HIF-1α, allowing it to translocate into the nucleus and directly up-regulate the transcription of multiple key glycolytic enzymes. In turn, this drives macrophage metabolic reprogramming from oxidative phosphorylation toward glycolysis. At the same time, the heightened glycolytic flux further inhibits mitochondrial respiratory-chain activity, disrupts the tricarboxylic-acid cycle, and promotes additional succinate accumulation, thereby establishing a succinate -HIF-1α-glycolysis positive-feedback loop that tightly couples metabolic state with inflammatory signaling (93–95). In addition, PKM2 dually regulates HIF-1α activity through direct binding to HIF-1α and modulation of succinate production. Succinate acts as a metabolic signal that enhances HIF-1α stability. During glycolysis, PKM2 catalyzes the conversion of phosphoenolpyruvate to pyruvate, with its enzymatic activity directly governing glycolytic flux. These mechanisms collectively drive the Warburg effect in macrophages (96), thereby intensifying intestinal crypt inflammation (97) and exacerbating the symptoms of inflammatory bowel disease.

**Figure 1:**
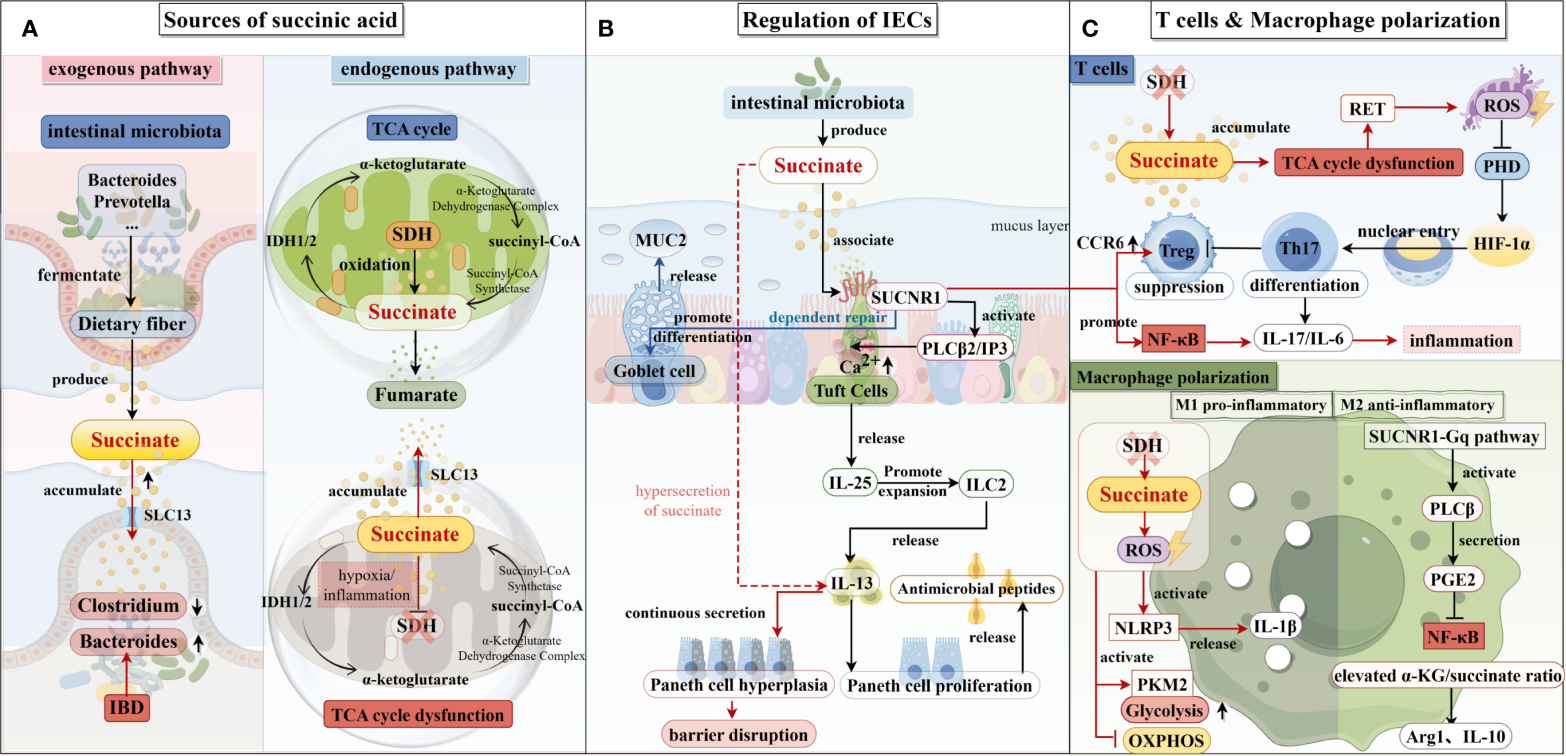
Succinate production pathways and their impact on intestinal epithelial cells, T cells, and macrophages. **(A)** The exogenous and endogenous pathways of succinate production are illustrated on the left. Succinic acid is predominantly produced in the intestine by Bacillus spp. and Prevotella spp. In patients with inflammatory bowel disease (IBD), dysbiosis of the intestinal microbiota—characterized by an increase in Bacteroides spp. and a decrease in Clostridium spp.—further promotes the growth of succinate-producing bacteria, leading to elevated succinate production. The primary endogenous pathway for succinate production is the tricarboxylic acid cycle, where succinyl-CoA is converted to succinate, which is subsequently converted to fumarate in the presence of succinate dehydrogenase (SDH). Under hypoxic conditions, SDH activity is reduced, which results in the accumulation of succinate. **(B)** In the center, succinate’s role in regulating intestinal epithelial cell function is depicted. Succinate produced by the intestinal microbiota binds to the SUCNR1 receptor on the surface of epithelial cells, stimulating the secretion of IL-25, which activates ILC2s, leading to the release of IL-13 and the induction of a type II immune response. This response increases the number of Paneth cells and promotes the secretion of antimicrobial peptides. Additionally, succinate enhances the release of mucin 2 from goblet cells, thus maintaining intestinal barrier function. **(C)** On the right, the effects of succinate on T cells and macrophages are shown. In T cells, decreased SDH activity promotes Th17 cell differentiation while inhibiting Treg cell differentiation, resulting in increased secretion of IL-17 and IL-6, which enhances the inflammatory response. In M1 macrophages, reduced SDH activity leads to succinate accumulation and increased production of reactive oxygen species (ROS), which activates downstream signaling pathways and increases IL-1β secretion, further promoting inflammation. In M2 macrophages, succinate enhances the ratio of α-KG to succinate via the SUCNR1-Gq pathway, stimulating the secretion of IL-10 and resolving the inflammatory response.

The dual roles of succinate indicate that its immunomodulatory effects are highly context- and concentration-dependent. Future studies must delineate the precise concentration thresholds and metabolic parameters governing the polarization switch between M1 and M2 macrophage phenotypes. This will enable achieving refined control over the balance between oxidative phosphorylation and glycolysis, thereby informing therapeutic strategies targeting succinate metabolism in inflammatory diseases such as IBD (**Figure 1**).

## 4 Succinic acid affects IBD by interacting with metabolites in the intestinal lumen

The pathogenesis of IBD is closely associated with disturbances in the intestinal metabolic microenvironment. As a key intermediate metabolite of the tricarboxylic acid cycle, succinic acid not only regulates inflammation via receptor-dependent and -independent pathways but also interacts with various intestinal metabolites—including bile acids, short-chain fatty acids (SCFAs), and tryptophan derivatives—which collectively influence intestinal barrier integrity, immune homeostasis, and microbial balance.

### 4.1 Bile acids

**Figure 2:**
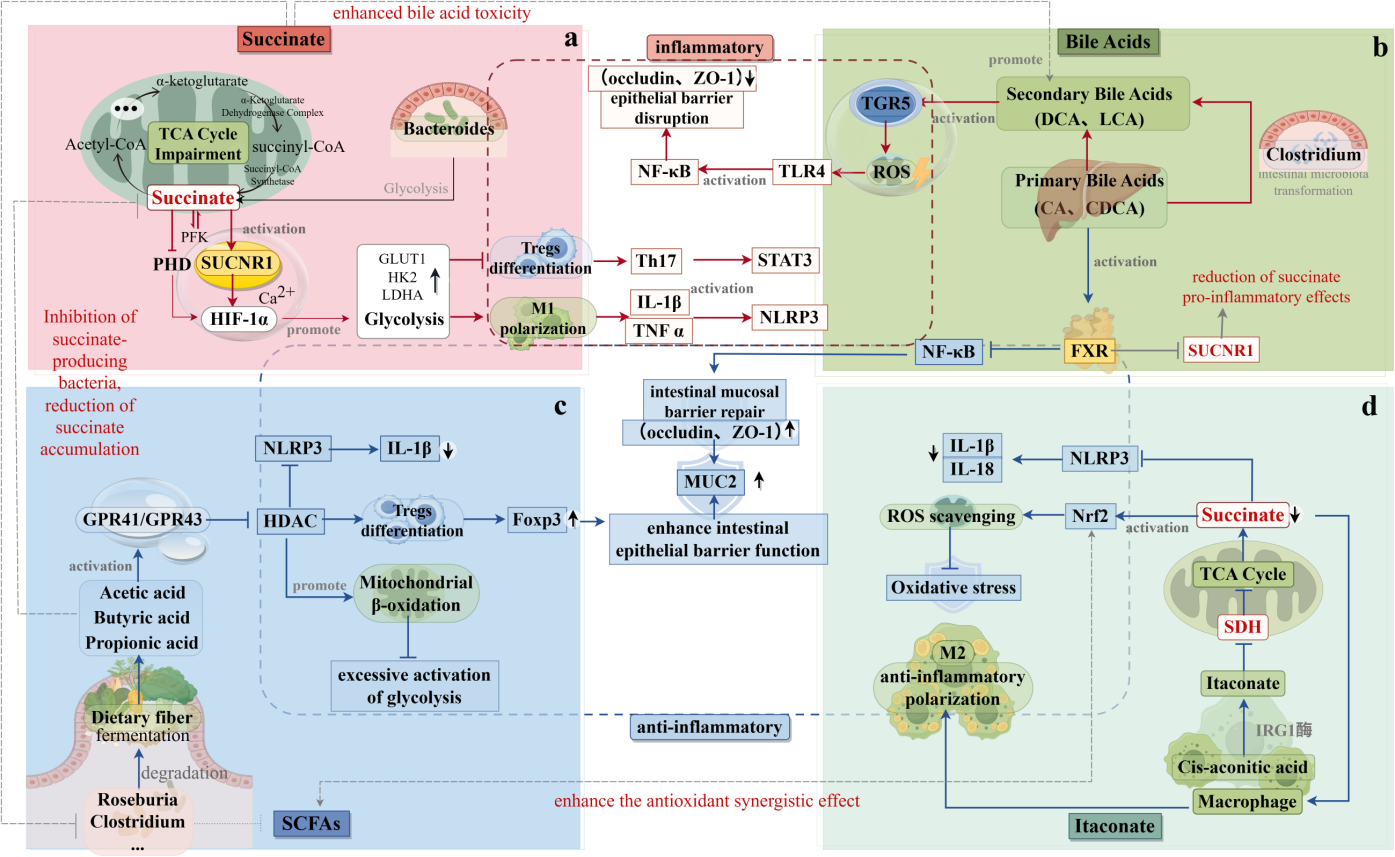
Interaction of Succinic Acid with Gut Flora Metabolites. **(a)** The metabolic origins and mechanisms of action of succinic acid are depicted in the upper left. Succinic acid inhibits the growth of butyrate-producing bacteria (e.g., Clostridium spp.), thereby reducing the production of short-chain fatty acids (SCFAs) while promoting the production of secondary bile acids (e.g., deoxycholic acid). This shift in metabolite production exacerbates epithelial damage. **(b)** The metabolic sources and mechanisms of action of bile acids are illustrated in the upper right. FXR signaling inhibits SUCNR1 expression, thereby reducing the pro-inflammatory effects of succinate. Additionally, secondary bile acids attenuate the Nrf2 antioxidant pathway and counteract the protective effects of itaconic acid, thereby exacerbating oxidative stress. **(c)** The metabolic sources and mechanisms of action of short-chain fatty acids are shown in the lower left. Butyric acid inhibits succinate-producing bacteria, reducing succinate accumulation. In addition, SCFAs activate the Nrf2 antioxidant pathway and synergize with itaconic acid to inhibit reactive oxygen species (ROS). **(d)** The metabolic origins and mechanisms of action of itaconic acid are depicted in the lower right. Itaconic acid inhibits succinate production by suppressing SDH, while enhancing the antioxidant synergistic effects of Nrf2 and SCFAs.

Li et al. demonstrated that dietary supplementation with succinic acid activated intestinal pro-inflammatory responses via the porcine cognate receptor SUCNR1, and subsequently, succinic acid-induced pro-inflammatory cytokines blocked the activation of the FXR and its target genes, affecting luminal bile acid transport, promoting hepatic bile acid secretion, and ultimately causing an impairment of enterohepatic recycling of bile acids. Changes in the composition of bile acids in the liver, ileum, and plasma suggested that dietary succinic acid induced porcine bile acid homeostatic imbalance (98). Conversely, Wang et al. found that mice receiving a succinate-enriched diet combined with the secondary bile acids ursodeoxycholic acid (UDCA) and lithocholic acid (LCA) showed significant improvements in hyperglycemia, hyperlipidemia, and hepatic steatosis. These findings suggest that succinate can beneficially modulate the bile acid profile, particularly in metabolic disorders such as obesity, to enhance intestinal barrier integrity and reduce inflammation (21) (**Figure 2**).

### 4.2 Short-chain fatty acids

Succinate and SCFAs are both critical microbial fermentation products that synergistically influence intestinal and systemic metabolic homeostasis. Succinate acts as a precursor for certain SCFAs; for example, specific gut bacteria convert succinate to propionate via intermediates such as succinyl-CoA, methylmalonyl-CoA, and propionyl-CoA (18). In addition, both succinate and SCFAs are able to influence intestinal gluconeogenesis (IGN) and glucose homeostasis through unique mechanisms. Succinic acid, as a direct substrate of IGN, participates in glucose synthesis and release into the portal vein by being absorbed by intestinal epithelial cells, while SCFAs (e.g. propionic acid and butyric acid) enhance intestinal barrier function and energy metabolism through activation of their receptors (e.g., GPR41 and GPR43) and indirectly promoting IGN activity (99). The synergistic effect of the two can stabilise systemic metabolism by improving the intestinal environment. For example, studies have shown that dietary supplementation with oligofructose not only increased intestinal succinate levels, but also promoted propionate synthesis, further enhancing IGN activity and glucose regulation (43, 44). In terms of barrier function, succinate modulates inflammatory signaling by activating the SUCNR1 receptor on immune cells, whereas short-chain fatty acids enhance mucus secretion and promote epithelial integrity to maintain barrier homeostasis (100). The synergistic effects of succinate and SCFAs are crucial for controlling intestinal inflammation and restoring microbial balance.

SCFAs also exert systemic effects through regulation of lipid metabolism, insulin sensitivity, and energy expenditure. While SCFAs stimulate hepatic lipid metabolism, succinate enhances these effects by regulating IGN and SUCNR1 signaling. Dynamic changes in succinate and SCFAs levels during inflammation can thus significantly impact gut microbial ecology and systemic metabolic stability (40). In summary, succinate and SCFAs are not only directly linked in metabolic pathways, but also regulate intestinal function, glucose homeostasis and systemic energy metabolism through synergistic effects. This metabolic interplay reveals the complex roles of gut microbial metabolites in health and disease and provides important insights for exploring novel metabolic disease intervention strategies.

Gut flora can produce short-chain fatty acids via the succinic acid pathway, for example, using succinic acid in the tricarboxylic acid cycle as a substrate, which is converted to propionic acid via several intermediates such as succinyl-coenzyme A, methylmalonyl-coenzyme A, and propionyl-coenzyme A. Propionic acid can regulate immune cell gene expression, differentiation, chemotaxis, proliferation and apoptosis, and thus participate in immune modulation. Numerous studies have found that propionic acid regulates the immune response through two main pathways: activation of free fatty acid receptors and inhibition of histone deacetylase, which suppresses the pro-inflammatory response regulated by nuclear transcription factor-κB and activator protein-1 (101, 102). Short-chain fatty acids such as propionic, acetic and butyric acids bind to free fatty acid receptors (GPR) distributed on the surface of a variety of cells, including intestinal epithelial cells, immune cells and endocrine cells, thereby triggering downstream signalling pathways and regulating immune cell proliferation, differentiation, chemotaxis, and inflammatory mediator production. For example, activation of GPR43 and GPR41 promotes chemotaxis and proliferation of peripheral immune cells and enhances the integrity of the mucosal barrier to a certain extent, thus maintaining the homeostasis of the intestinal microenvironment (103, 104). In contrast, another pathway suggests that propionic acid and butyric acid can inhibit the activity of histone deacetylases (HDACs), which in turn affects the degree of chromatin opening and the level of gene transcription. This process down-regulates the activation of transcription factors such as nuclear transcription factor-κB (NF-κB) and activator protein-1 (AP-1), which inhibits the expression of pro-inflammatory factors and attenuates excessive inflammatory responses. At the same time, the regulation of HDACs activity can also affect T cell subpopulation differentiation and the establishment of immune tolerance, which helps to maintain the body’s immune homeostasis against self-antigens and foreign harmless antigens (105, 106). In conclusion, intestinal flora synthesise SCFAs such as propionic acid, butyric acid and acetic acid using substrates such as succinic acid, and these metabolites are capable of exerting a profound effect on the host immune system through a variety of mechanisms such as binding to free fatty acid receptors and inhibiting the activity of HDACs. In summary, SCFAs derived from microbial metabolism of succinate play multifaceted roles in immune modulation, epithelial protection, and systemic metabolic regulation, emphasizing the importance of the succinate–SCFAs axis in maintaining intestinal and whole-body health.

### 4.3 Itaconic acid

Itaconic acid (ITA) is a valuable biochemical currently produced industrially by Aspergillus terreus from glucose and starch (107), and it is also endogenously synthesized in activated macrophages of higher eukaryotes (108). It has been found that an increase in ITA can lead to the accumulation of citric and succinic acids in activated immune cells such as M1-type macrophages (109). Mechanistically, it is suggested that ITA can competitively inhibit SDH activity, thereby reducing succinate oxidation and thus inhibiting RET-induced inflammatory responses (110). Related experiments have shown that ITA acts as a mitochondrial metabolite, which exhibits inhibitory activity against SDH, which in turn leads to mitochondrial dysfunction (111). However, inhibition of SDH by ITA also causes mitochondrial dysfunction, suggesting a complex regulatory feedback loop between these metabolites. In the early phase of inflammation, inhibition of SDH elevates succinate and ITA, which stabilize HIF-1α and promote IL-1β expression. As inflammation resolves, HIF-1α upregulates pyruvate dehydrogenase kinase (PDK), which inhibits pyruvate dehydrogenase, resulting in decreased levels of succinate and ITA, and restoring metabolic balance (111–113). Thus, the dynamic balance between metabolites allows cells to better perform their physiological functions and better maintain the homeostasis of the internal environment. It has been found that succinic acid can interact with intestinal luminal metabolites such as bile acids, short-chain fatty acids, and ITA, which can work together to maintain intestinal homeostasis. However, the related studies are still relatively scarce, which also provides a direction for the later experiments.

## 5 Targeting intervention in succinate metabolism to explore the prospects of treating inflammatory bowel disease

To date, studies on the relationship between succinate metabolism and inflammation have proliferated and have demonstrated that succinate plays an important role in inflammatory diseases such as hepatitis and arthritis. Dysregulation of succinic acid metabolism has been closely associated with a variety of diseases, ranging from metabolic syndrome to inflammatory diseases, and has been investigated in a number of diseases including inflammatory bowel disease (14), diabetes mellitus (114), retinal complications (115), cardiovascular disease (116), fatty liver (117), rheumatoid arthritis (118), sepsis (119), obesity (59) and cancer (120) multiple diseases. Targeted intervention in succinate metabolism represents a promising therapeutic strategy for inflammatory bowel disease.

### 5.1 Targeting the SUCNR1 receptor: development of antagonists

SUCNR1, as a key molecule for metabolite sensing, is a common driving factor for the progression of various metabolic diseases. In obesity, SUCNR1 activation promotes macrophage infiltration into adipose tissue, contributing to insulin resistance and poor glucose tolerance—hallmarks of type 2 diabetes (121). Similarly, succinate accumulation has been implicated in the progression of atherosclerosis by activating SUCNR1 and promoting smooth muscle cell phenotypic transformation, macrophage polarization, and endothelial dysfunction (122). Succinate accumulates in areas of inflammation and modulates immunity through SUCNR1. Patients with CD have elevated serum and intestinal succinate levels and increased SUCNR1 expression in intestinal tissues and fibroblasts, which in turn mediates the expression of pro-inflammatory factors in macrophages and enhances intestinal inflammation (11). Thus, SUCNR1 signalling can exacerbate inflammation and fibrosis, and intervention from the succinate-SUCNR1 axis is also an important target for improving the inflammatory response in CD patients.

### 5.2 Regulation of flora and metabolites: probiotics and dietary interventions

Modulating the intestinal microbiota and its metabolites presents another promising avenue. For example, administration of 0.08% erythromycin has been shown to downregulate fecal succinate levels, thereby reducing inflammation and oxidative stress while maintaining gut homeostasis (123). In necrotizing enterocolitis (NEC), elevated fecal succinate levels were associated with disease severity, and succinate-mediated activation of the HIF-1α pathway was implicated in disease progression (14). Herbal therapies have also demonstrated efficacy in regulating succinate levels. The traditional Chinese medicine formula *Xianglian Pill* was shown to reduce microbial succinate production by modulating the PHD2/HIF-1α signaling axis. This, in turn, promoted regulatory T cell (Treg) differentiation and alleviated colitis symptoms in a rat model of ulcerative colitis (15).

### 5.3 Metabolic pathway intervention: SDH inhibitors and HIF-1α regulation

In recent years, it has also been found that inhibition of SDH with dimethylmalonate, which slows down the rate of succinate oxidation in macrophages, significantly inhibits the generation of ROS from complex I, leading to a reduction in LPS-induced HIF-1α expression and IL-1β production, a result that suggests that SDH may also be an important target for intervening in inflammatory responses. Curcumin has been shown to reduce succinate accumulation by counteracting fatty acid oxidation and to ameliorate chronic inflammation and fibrosis in the liver by preventing primary hepatic stellate cell activation in mice by blocking the succinate/HIF-1α signalling pathway (124). Cinnamaldehyde has been shown to suppress HIF-1α signaling by preventing succinate accumulation in macrophages, exerting anti-inflammatory effects in rheumatoid arthritis models (125). Moreover, SDH inhibition can block the HIF-1α/VEGF signaling axis, thereby reducing angiogenesis and attenuating inflammation (118).

These findings suggest that modulation of metabolic enzymes and signaling molecules involved in succinate pathways can have broad anti-inflammatory and disease-modifying effects.

## 6 Summary and discussion

This review systematically elucidates the multidimensional regulatory role of succinate in inflammatory bowel disease (IBD) and introduces the novel concept of the “succinate metabolic loop”, positioning succinate as a central node within the metabolism–immunity–microbiota triad. Mechanistically, we demonstrate that dynamic crosstalk between endogenous and exogenous succinate orchestrates intestinal barrier integrity, immune-cell polarization, and microbial homeostasis via SUCNR1 signaling, HIF-1α stabilization, and metabolic reprogramming. Moreover, we propose that succinate, short-chain fatty acids, and itaconate constitute a dynamic “metabolic tug-of-war” that co-shapes Th17/Treg balance through the HDAC–NF-κB axis. We further reveal the spatiotemporal duality of SUCNR1 signaling—early tissue repair versus late fibrogenesis—thereby providing a theoretical foundation for stage-specific precision therapy in IBD. We highlight the triple role of the “succinic acid metabolic circuit” as a central node connecting host metabolism, immune response and microbial homeostasis in inflammatory bowel disease (IBD). Despite the significant progress, the following key issues still need to be explored in depth: 1) Establishing succinate concentration thresholds: The sensitivity to succinate varies across cell types, including epithelial cells and macrophages. It is essential to determine the functional concentration thresholds by mapping the three-dimensional succinate gradient within the intestinal microenvironment. Techniques such as microfluidics and spatial metabolomics will be instrumental in clarifying the transition points between succinate’s protective and pathogenic roles; 2) Spatiotemporal dynamics of SUCNR1 signaling: Understanding SUCNR1’s role across the disease timeline requires single-cell transcriptomic profiling of SUCNR1^+^ cell subsets during various IBD stages (initiation, progression, resolution). Integration with conditional knockout models will help validate SUCNR1’s dynamic functions and clarify its role in inflammation regulation and tissue remodeling; 3) Translational challenges and therapeutic opportunities: Although SUCNR1 antagonists have shown promising anti-inflammatory effects in animal models, their potential to inhibit intestinal epithelial regeneration warrants further investigation. Organoid–immune cell co-culture systems can provide valuable insight into the balance between therapeutic efficacy and tissue repair. Additionally, microbiome-targeted interventions—such as engineered succinate-consuming probiotics—offer a novel strategy to modulate inflammation safely and precisely by reprogramming the gut metabolite network. By redefining succinate as a central player in the IBD metabolic–immune–microbial axis, this review offers new perspectives on disease pathogenesis and identifies novel therapeutic targets. Continued research into succinate’s concentration-dependent, temporal, and spatial dynamics will pave the way for personalized metabolic interventions and microbiota-based therapies in IBD and other inflammatory disorders.
